# Adult‐onset Still's disease in Western Australia: Epidemiology, comorbidity and long‐term outcome

**DOI:** 10.1111/1756-185X.14424

**Published:** 2022-08-24

**Authors:** Johannes Nossent, Warren Raymond, Helen Keen, David B. Preen, Charles A. Inderjeeth

**Affiliations:** ^1^ Department of Rheumatology Sir Charles Gairdner Hospital Perth Western Australia Australia; ^2^ Rheumatology Group, School of Medicine University Western Australia Perth Western Australia Australia; ^3^ Department of Rheumatology Fiona Stanley Hospital Perth Western Australia Australia; ^4^ School of Population and Global Health Perth Western Australia Australia

**Keywords:** adults, comorbidity, epidemiology, lookback, Still's disease, survival

## Abstract

**Aim:**

Adult‐onset Still’s disease (ASD) is a rare, potentially life‐threatening autoinflammatory condition. As reported prevalence shows regional variation and long‐term outcome data are scarce, we investigated epidemiology and long‐term health outcomes of ASD in Western Australia (WA).

**Methods:**

Population‐based cohort study using longitudinally linked administrative health data from all WA hospitals between 1999 and 2013 for ASD patients (ICD‐10‐AM M06.1) and controls matched for age, gender, and index year. Rate ratios and odds ratios (RR/OR) with 95% confidence intervals (CI) compared ASD patients with controls.

**Results:**

The average ASD incidence (n = 52) was 0.22/100 000 with 2.4/100 000 point‐prevalence as of December 31, 2013. ASD patients (median age 41.5 years, 59.6% female) had higher odds of previous liver disease (OR 2.67, 95% CI 1.31‐5.45), fever (OR 54.10, 95% CI 6.60‐433.0), rash (OR 15.70, 95% CI 4.08‐60.80), and serious infections (OR 4.36, 95% CI 2.11‐22.80) than controls. Despite biological disease‐modifying antirheumatic drugs in 27% of patients, ASD patients had higher odds for joint replacement (n = 7, 13.5%) (OR 45.5, 95% CI 4.57‐93.70), osteoporosis (OR 31.3, 95% CI 3.43‐97), and serious infections (RR 5.68; 95% CI 6.61‐8.74) during follow up. However, crude mortality (11.5% vs 7.5%; *P* = 0.34), survival at 1 and 5 years (*P*= 0.78), and last modified Charlson Comorbidity score (median 2 vs 2) were similar between groups.

**Conclusion:**

The epidemiology and demographics of ASD in Western Australia fall within the internationally reported range. ASD patients present increased rates of liver disease, rash, and serious infections before disease onset. Mortality following ASD was not increased for 5 years despite high rates of chronic arthritis requiring joint replacement, serious infections, and osteoporosis.

## 1 INTRODUCTION

Still's disease (SD) is a rare systemic inflammatory disease of unknown origin, first described in children by Sir George Still more than a century ago.^1^ Bywaters et al described in 1971 a series of adult women with clinical features reminiscent of SD and proposed the term adult‐onset Still's disease (ASD).^2^ ASD has reported prevalence ranges from 1 to 34 cases per million^3^, ^4^, ^5^ and typically presents with a mixture of symptoms where daily fever spikes, sore throat, evanescent rash, and polyarthritis in the presence of neutrophilia and elevated acute‐phase reactants are the most frequent findings.^3^ However, myalgia, lymphadenopathy, fulminant hepatitis, serositis, consumption coagulopathy, and myocarditis can also occur.^6^, ^7^, ^8^ ASD is a diagnosis of exclusion and, as a result, is usually only considered when treatment of suspected infections has been unsuccessful.^3^, ^6^ ASD is classified as an autoinflammatory disease where a complex interplay of genetic, infectious, and other environmental factors trigger overproduction of proinflammatory cytokines, which drive the clinical manifestations and ultimately can lead to the life‐threatening macrophage activation syndrome (“cytokine storm”).^7^, ^8^ The clinical course of ASD is unpredictable because it appears self‐limiting in some patients but leads to recurrent exacerbations of systemic inflammation and/or the development of chronic deforming arthritis in many others.^9^, ^10^, ^11^ With scarce data available from Australasia, we investigated the epidemiological characteristics, previous conditions, and long‐term clinical outcomes in patients hospitalized for ASD in Western Australia (WA) over a 14‐year period.

## 2 MATERIALS AND METHODS

### 2.1 Data sources

Data were derived from the WA Rheumatic Disease Epidemiological Registry (WARDER) that contains routinely collected longitudinal linked health data for patients with rheumatic diseases from hospitals for the entire state of WA as described elsewhere.^12^, ^13^ Sourced from the Hospital Morbidity Data Collection, WA Cancer Registry, WA Mortality Registry or the Emergency Department Data Collection in the state of WA (population 2.5 million) these datasets are linked through a validated process of probabilistic matching and clerical review to provide deidentified individual longitudinal health data over the period 1980‐2015. The final data set contained sociodemographic data, all principal and secondary diagnoses for all earlier and subsequent hospital contacts for each participant, information on principal and secondary procedures performed, length and type of admission (eg, intensive care) in addition to diagnostic codes for any ED visit, ever‐recorded cancer type, and time and cause of death during the observation period. WARDER also contains a large age‐ and sex‐matched “comparator” group of patients selected from the WA Electoral Roll on the basis of requiring hospital care in the study period but not having a registered diagnostic code for rheumatic disease in any data collection linked through the WA Data Linkage System (WADLS).

### 2.2 Study cohort

For this population‐level observational study we included persons over 16 years of age with a recorded first diagnosis of ASD (International Statistical Classification of Diseases 10^th^ revision Australian modification [ICD‐10‐AM] M06.10‐M06.19) while residing in WA between January 1999 and December 2013. We excluded patients with possible ASD before 1999 because no specific coding for ASD was available in the ICD‐9‐Clinical modification with code 714.2 “Other rheumatoid arthritis with visceral or systemic involvement” considered too ambiguous. In an earlier study, ICD‐10 coding for ASD was found to have 83% positive predictive value for a clinical diagnosis of ASD and 78% sensitivity for fulfillment of the Yamaguchi criteria.^14^ For this study, each ASD patient was matched with up to five controls matched for age and gender but also for the same year of requiring hospital admission as the incident ASD case. WARDER controls are not healthy controls because they required hospitalization for a wide range of indications other than inflammatory rheumatic disease (see Figure S1), but survival in WARDER controls has been shown to be similar to that in the general population.^15^ Date and primary causes of death were extracted from the WA Death Registry.

### 2.3 Outcome ascertainment

As the first hospital contact was not necessarily for ASD, we defined a time‐zero (*T*_0_), which for ASD patients was the date of ASD diagnosis and for each control the date that most closely mirrored *T*_0_ for the matched ASD patient. We defined the lookback period as all observation time before *T*_0_ and follow up as all observation time more than 30 days after *T*_0_. We defined the occurrence of serious infections as episodes leading to presentation at the Emergency Department and/or hospital admission resulting in an infectious disease code.^13^ Study measures were the presence of specific ASD manifestations (Table S1) and the documented accrual of organ‐system‐specific, as well as overall weighted, comorbidity before and after T_0_ according to the validated and prognostically important Charlson comorbidity index (CCI), in‐hospital mortality, re‐admission rate within 30 days, and survival at 1 and 5 years of *T*_0_.

### 2.4 Statistical analyses

Descriptive statistics are presented as median plus interquartile range (IQR) for numeric variables and proportions for categorical variables, unless otherwise indicated. Historical population data for WA were obtained from the Australian Bureau of Statistics (https://www.abs.gov.au/statistics/people/population/national‐state‐and‐territory‐population/latest‐release#data‐downloads‐data‐cubes). Average annual incidence and point prevalence rates are given per 100 000 population with the total number of cases as numerator and a denominator based on the adult population in that year. A generalized log‐linear regression model (Poisson) was used to analyze the trend in the number of cases per year. Differences for numeric results were compared by non‐parametric methods (Kruskal‐Wallis) and for proportions by χ^2^ test with Yates correction where needed. All‐cause hospitalization and Emergency Department visit rates (expressed as number per 100 person‐years at risk) and odds for comorbidity/complications in ASD patients and controls were compared by conditional maximum likelihood estimates of odds ratios (OR) and rate ratios (RR) with 95% confidence intervals (CI). Kaplan‐Meier estimates were used to compare survival between ASD and control with *P* values presented from the log‐rank test. Analyses were performed using SPSS v27.0 software (IBM, Armonk, NY, USA) with two‐sided *P*‐values less than 0.05 considered to be statistically significant.

### 2.5 Ethics

This project was approved by the Human Research Ethics Committee at the WA Department of Health (HREC 2016.24) with the condition to prevent potential identification by confidentializing small numbers (n < 5).

## 3 RESULTS

**FIGURE 1:**
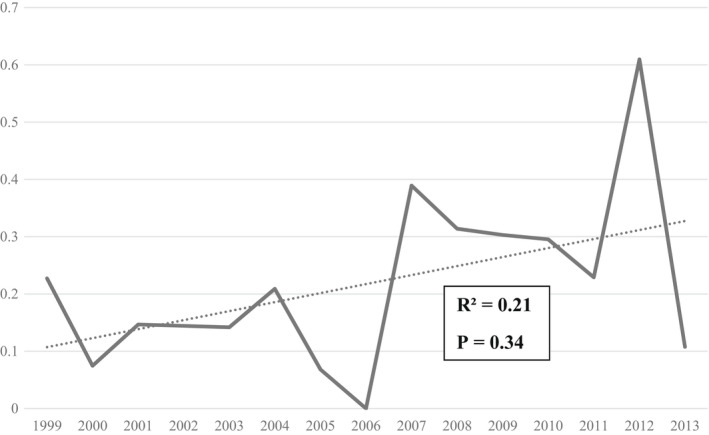
Annual incidence rate with trendline of adult‐onset Still's disease in Western Australia per 100 000 population over the study period

There were 52 incident cases of ASD in the study period, comprising 31 females (59.6%, median age 42 years) and 21 males (40.4%, median age 39 years). The average annual incidence for ASD was 0.22/100 000 (95% CI 0.14‐0.32) and did not change significantly (*R* ^2^ = 0.21, *P* = 0.34) over the 14‐year period (Figure 1). With 46 surviving patients the point‐prevalence of ASD on December 31, 2013 was 2.4 per 100 000 (95% CI 1.79‐3.2).

Demographic and previous medical details of the ASD patients (Table 1) showed few gender differences regarding age at onset, regional presentation, or insurance status. Median length of hospital stay for first ASD presentation was 9 days (IQR 4.5‐21.5) with some patients (n < 5, 5.7%) requiring intensive care unit admission. ASD‐related complications included acute kidney injury (7.7%), consumption coagulopathy (7.7%), serositis (5.8%), macrophage activation syndrome (MAS) (1.9%), and in‐hospital mortality (1.9%), with no significant differences between male and female patients (Table S2).

**TABLE 1:** Patient characteristics at first ASD presentation

	All (n = 52)	Female (n = 31)	Male (n = 21)	*P* value
Age onset (years)	41.5 (31‐56)	42 (32‐51)	39 (25‐64)	0.64
Indigenous background	<5 (1.9)	<5 (3.2)	0	NA
Country of birth				
Australia	39 (75%)	24 (77.4%)	15 (71.4%)	
Europe	6 (11.5%)	<5 (12.9%)	<5 (9.5%)	
Asia	6 (11.5%)	<5 (6.4%)	<5 (19%)	
Africa	<5 (2.5%)	<5 (3.2%)	‐	
Hospital location				
Metropolitan	44 (85%)	26 (84%)	18 (86%)	0.73
Regional	8 (15%)	5 (16%)	<5 (14%)	
Admitted from Emergency Department	28 (54%)	16 (52%)	12 (57%)	0.25
Insurance status				
Public	35 (67%)	21 (68%)	14 (67%)	0.96
Private	17 (33%)	10 (33%)	7 (33%)	
Complications observed				
Acute renal failure	<5 (7.7%)	<5 (3.2%)	<5 (14.3%)	0.17
Consumption coagulopathy	<5 (7.7%)	<5 (6.4%)	<5 (9.5%)	0.39
Serositis	<5 (7.7%)	<5 (3.2%)	<5 (14.3%)	0.17
MAS/ARDS	<5 (1.9%)	0	<5 (4.7%)	‐
Procedures performed				
Skin biopsy	6 (11.5%)	<5 (12.9%)	<5 (9.5%)	0.45
BM biopsy	8 (15.4%)	5 (16.1%)	<5 (14.3%)	0.78
Liver biopsy	<5 (5.8%)	0	<5 (9.5%)	‐
Lymph gland biopsy	<5 (1.9%)	<5 (3.2%)	<5 (4.7%)	0.66
Arthrocentesis	10 (19.2%)	6 (19.4%)	<5 (19%)	0.91
LOS (days)	9 (4.5‐21.5)	8 (5‐11)	11 (3‐26.5)	0.17
Required ICU admission	<5 (5.8%)	<5 (3.2%)	<5 (9.5%)	0.28

*Note*: Small numbers are given as <5 because of Human Research Ethics Committee requirements to prevent identification. Data represent median values (interquartile range) or frequency (%)

Abbreviations: ARDS, acute respiratory distress syndrome; ASD, adult‐onset Still’s disease; BM, bone marrow; ICU, intensive care unit; LOS, length of stay; MAS, macrophage activation syndrome.

During a median lookback period of 203 months (IQR 40‐280), ASD patients had higher rates/100 person‐years for hospital admission and ED presentations before diagnosis than controls as well as higher odds of being diagnosed with liver disease, fever of unknown origin, rash, and serious infections (Table 2). Forty‐eight ASD patients (92.3%) had at least one hospital contact in the year before diagnosis. Most ASD patients (89.5%) and controls (96.9%) had recorded comorbidity before *T*_0_ and although median CCI scores were similar between the groups, slightly more ASD patients had multimorbidity (m‐CCI ≥2) (Table 2).

**TABLE 2:** Patient characteristics and previous medical conditions in ASD patients and controls during lookback period

	ASD	Controls	RR/OR (95% CI)	*P* value
Age at first hospital contact (years)	25 (16‐40.5)	25 (17‐41)	‐	0.85
Lookback (months)	203 (40‐280)	190 (73‐260)	‐	0.81
Total person‐years	752	3211	‐	‐
ED visit rate/100 person‐years	29.4 (25.7‐33.5)	11.5 (10.3‐12.6)	2.42 (1.25‐4.91)	0.007
Admission rate/100 person‐years	79.5 (62.9‐97.9)	47.4 (36.2‐61.3)	1.71 (1.2‐2.5)	0.003
Previous diagnoses				
Chronic pulmonary disease	46 (88.5)	219 (96.1)	0.32 (0.11‐0.93)	0.04
Liver disease	15 (28.8)	30 (13.2)	2.67 (1.31‐5.45)	0.01
Cardiovascular event	<5 (3.8)	15 (6.6)	0.57 (0.13‐2.64)	0.75
Diabetes mellitus	8 (3.5)	84 (7.7)	0.31 (0.13‐0.68)	0.02
Cancer	<5 (5.8)	19 (8.3)	0.67 (0.19‐2.37)	0.77
Peptic ulcer disease	<5 (7.7)	<5 (1.8)	4.67 (1.13‐19.32)	0.04
Renal disease	<5 (5.8)	6 (2.6)	2.27 (0.55‐9.38)	0.38
FUO	10 (19.2)	<5 (0.4)	54.1 (6.6‐433.4)	<0.001
Rash	9 (17.3)	<5 (1.3)	15.7 (4.08‐60.8)	<0.001
Sore throat	<5 (3.8)	0 (0)	NA	NA
Serious infection	8 (15.4)	10 (4.4)	4.36 (2.11‐22.8)	0.001
m‐CCI score at time zero	1 (1‐2)	1 (1‐2)	‐	0.68
m‐CCI = 0	6 (11.5%)	7 (3.1)	‐	0.02
m‐CCI = 1	27 (51.9%)	153 (67.1)		
m‐CCI ≥2	19 (36.5%)	68 (29.8)		

*Note*: Small numbers are given as <5 because of Human Research Ethics Committee requirements to prevent identification. Data represent median values (interquartile range) or frequency (%) with rate ratio and odds ratios (95% confidence intervals).

Abbreviations: ASD, adult‐onset Still’s disease; CI, confidence interval; ED, Emergency Department; FUO, fever of unknown origin; m‐CCI, modified Charlson comorbidity Index (without the rheumatic disease); OR, odds ratio; RR, rate ratio.

**FIGURE 2:**
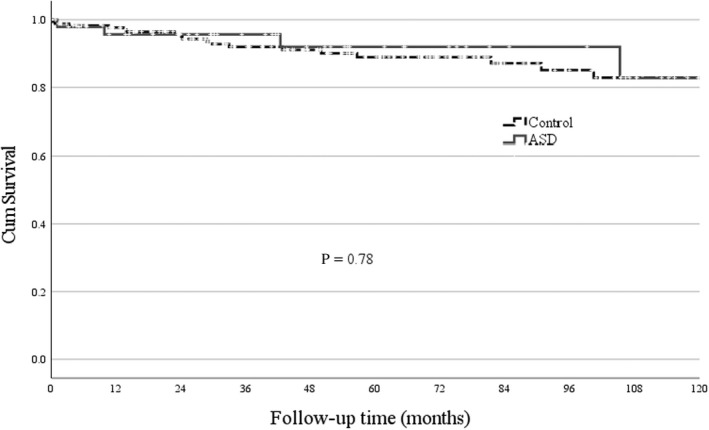
Kaplan‐Meyer curves and log‐rank test result for survival beyond 30 days in adult‐onset Still's disease patients and age‐/gender‐matched hospitalized controls

Readmission within 1 month after discharge was more frequent in ASD patients than controls (19.2% vs 9.6%, *P* = 0.003) with five out of ten ASD patients readmitted with disease flare as the primary diagnosis. During a median follow up of 49 months (IQR 24‐84) a total of 36 ASD flares occurred in 13 (25%) patients for a flare rate of 14.7/100 person‐years (Table 3). Forty‐eight ASD patients and 177 controls required hospital care beyond 30 days of follow up (94% vs 78%, respectively; OR 3.45, 95% CI 1.27‐11.7) with higher rates for both admission and Emergency Department visits in ASD patients (Table 3). Among these ASD patients a total of 13/48 (27%) received intravenous biological disease‐modifying antirheumatic drug therapy, of which seven patients (13.5%) underwent joint replacement surgery after a median period of 77 months (IQR 64‐103) with total hip (40%) and knee (40%) replacement the most frequent procedures. Osteonecrosis (ICD‐10 code M87) was diagnosed in a 51 year old female patient 6 months after ASD diagnosis and in a 62 year old male patient >10 years after ASD diagnosis but no formal diagnosis of steroid‐induced osteonecrosis (Y42.0) was recorded. The overall frequency (27% vs 8%, *P* < 0.01) and the rate of serious infections per 100 person‐years (20 vs 3.6; RR 5.68, 95% CI 6.61‐8.74) remained increased for ASD patients over the observation period (Table 3). There was however no significant difference between ASD patients and controls in crude mortality (6/52, 11.5% vs 18/228, 7.5%; *P* = 0.34) or survival rates over 5 years (*P* = 0.78) (Figure 2). Adjusting for the baseline presence of DM or CVD did not significantly influence survival rates between patients and controls (log‐rank *P* = 0.74 and *P* = 0.82, respectively). The most frequent cause of death was malignancy in ASD patients (n = 6) (melanoma in two, metastasis of unknown primary in one with no registered cases of lymphoproliferative malignancies) and cardiovascular events in controls (n = 18) (Figure S2).

**TABLE 3:** Frequency of new complications registered in ASD patients and controls during follow up period.

	ASD (n = 48)	Controls (n = 177)	OR (95% CI)	*P* value
Follow up period (months)	42 (22‐80)	49 (24‐89)	‐	0.51
Total person years	245	927	‐	‐
30‐day readmission	10 (19.2)	15 (6.6)	3.52 (1.41‐8.40)	0.003
ASD flare rate (n = 36)	14.7 (10.5‐20.1)	‐	‐	‐
Serious infections rate	20 (14.8‐26.4)	3.6 (2.5‐5.0)	5.68 (3.61‐8.74)	<0.01
Subsequent diagnoses				
Joint replacement surgery	7 (13.5)	0	45.5 (4.57‐93.1)	<0.001
Osteoporosis	5 (9.6)	0	31.26 (3.43‐97)	<0.001
Chronic pulmonary disease	45 (93.7)	154 (87.0)	2.24 (0.6‐7.81)	0.31
Liver disease	16 (20.8)	27 (15.3)	2.78 (1.33‐5.74)	0.007
Cardiovascular event	7 (14.6)	15 (8.5)	1.86 (0.71‐7.84)	0.27
Diabetes mellitus	<5 (2.1)	<5 (1.1)	1.86 (0.51‐2.16)	0.52
Cancer	<5 (8.3)	10 (5.9)	1.51 (0.79‐5.87)	0.51
Peptic ulcer disease	<5 (2.1)	<5 (0.6)	3.75 (0.23‐60.9)	0.38
Renal disease	<5 (6.2)	9 (5.1)	1.24 (0.33‐4.78)	0.72
ED visit rate	105 (93‐120)	49 (44‐54)	2.15 (1.85‐2.50)	<0.01
Admission rate^a^	168 (151‐184)	58 (54‐63)	2.87 (2.52‐3.26)	<0.01
Median m‐CCI at last obs. Comorbidity score	2 (1‐2)	2 (1‐2)	‐	0.11
m‐CCI at last obs. =0	9	52	‐	0.24
m‐CCI at last obs. =1	32	109		
m‐CCI at last obs. ≥2	7	16		

*Note*: Data represent median values (interquartile range), frequency (%) or rate/100 person‐years with odds ratios presented with 95% confidence intervals.

Abbreviations: ASD, adult‐onset Still’s disease; CI, confidence interval; ED, Emergency Department; m‐CCI, modified Charlson comorbidity Index (without the rheumatic disease); OR, odds ratio.

^a^ Excluding admissions for drug infusions. Small numbers are given as <5 because of Human Research Ethics Committee requirements to prevent identification.

## 4 DISCUSSION

In this population‐based study stretching over 14 years, the average annual incidence of ASD in WA was 0.22/100 000, while point‐prevalence at December 31, 2013 was 2.4/100 000 and ASD was associated with 1.9% in‐hospital mortality. Compared with matched controls, ASD patients had significantly higher odds of serious infections and liver disease pre‐diagnosis, but survival during follow up was similar for both groups, despite an ASD flare rate of 14.7/100 person‐years and increased odds for chronic arthritis, joint replacement surgery, osteoporosis, and serious infections in ASD patients.

The ASD incidence based on case series varies from 0.16/100 000 in France to 0.4/100 000 in Norway, and a questionnaire‐based survey in Japan in 1993 found 144 ASD patients (87% fulfilled classification criteria) and estimated the national incidence at 0.28/100 000 and prevalence at 1.1/100 000.^4^, ^5^, ^16^ Using population‐wide hospital admission data, we found an annual incidence (0.22) and prevalence (2.4) of ASD that was comparable with a recent population‐based study from Poland using hospital discharge records between 2009 and 2018 that estimated annual incidence rate at 0.32/100 000 and point‐prevalence of 2.7/100 000 by 2019.^17^ With both age at diagnosis (42.5 years) and the female predominance (59.6%) also in the middle of the range reported in the literature, our findings indicate that ASD remains a relatively uncommon condition in WA for which there does not appear to be a substantial difference based on region or over time.^4^, ^5^, ^16^, ^17^, ^18^

ASD remains a diagnosis of exclusion as exemplified by the high number of procedural investigations performed during ASD admission to exclude other causes of disease manifestations and the relatively prolonged length of hospital stay of 9 days, which is higher than the 6 days reported from the USA.^19^ ASD‐related complications including kidney failure, consumption coagulopathy, and hemophagocytic lymphohistiocytosis/MAS were observed infrequently in line with findings from other studies,^17^, ^18^ although higher complications rates are reported in studies from dedicated/specialized centres.^20^, ^21^, ^22^ As a result, in‐hospital mortality was relatively low at 1.9% comparable with data from the USA and Poland,^17^, ^18^ but much lower than the 16% reported from Italy, mainly due to a high number of patients with fatal MAS.^21^

Baseline comorbidity scores (without the rheumatic disease component, because this was excluded in controls) at ASD diagnosis were not higher than in non‐ASD controls in our study, although the average CCI scores in the US survey, which included the score for rheumatic disease, were reportedly higher in ASD patients than controls (1.33 vs 0.81).^18^ The specific comorbid conditions that could potentially predispose to ASD in this study were a higher rate of previous serious infections and existing liver disease. Although infections have long been considered a trigger for ASD in (genetically) predisposed individuals,^23^, ^24^ we did not find evidence of an increased rate of specific serious infections, including pneumonia, sepsis, or bacteremia, urinary tract infection, and skin and soft‐tissue infections. As almost 95% of all serious infections had microbiological confirmation, it is less likely that ASD disease flares were misdiagnosed as serious infections, but it has been suggested that a particular combination of pathogenic microorganisms with genetic susceptibility (variations in, for example, human leukocyte antigen or cytokine genes) are likely essential determinants of ASD development and severity.^23^, ^25^ The prevalence of chronic lung disease over a nearly 20‐year period before diagnosis was high although not different for ASD patients and controls. The relevant CCI codes include conditions such as bronchitis, smoking, asthma, and emphysema, which are not specific for ASD and are frequent diagnoses in hospitalized patients. Although abnormal liver enzymes are a recognized feature of ASD, it is unclear whether the higher rate of pre‐existing liver disease in ASD patients versus controls (28.8% vs 13.2%) reflected impending ASD or classifies as a specific risk factor that will require further study.

There are limited data on long‐term survival in ASD.^26^ Crude mortality was 11.5% over a median follow up of 4.2 years in this study, which falls within the 25% mortality rate (n = 2 of 8) from France after more than 10 years of follow up, the 9.8% mortality rate over 3.9 years of follow up reported from a single center study in China, and the 5.4% mortality rate over 2.8 years from Japan.^5^, ^10^, ^16^, ^26^, ^27^ Crude mortality rates may be influenced by other population‐specific and comorbid factors, but the inclusion of a matched cohort of hospitalized controls provided comparative data demonstrating that overall survival up to 5 years was not worse for ASD patients compared with other hospitalized patients. Cardiovascular death was less and cancer‐related death was more frequent in ASD patients compared with controls. All cancers in ASD patients were solid organ malignancies and although not significant because of low numbers/statistical power, this supports the need for monitoring for the possibility of malignancy‐associated ASD.^28^, ^29^

Joint and bone complications were the main long‐term problems in up to 30% of ASD patients. The risk of chronic erosive arthropathy in a subgroup of ASD patients has long been recognized and despite the empirical use of biological drug therapy in 27% of patients a relatively large number of patients in this study (n = 7, 13.5%) required joint replacement surgery for symptomatic relief in the long run.^30^, ^31^ ASD treatment is deduced from observational studies and although randomized controlled trials to support the therapy of choice in ASD patients are lacking there is increasing evidence that cytokine targeting treatment is beneficial.^32^, ^33^, ^34^, ^35^ As detailed medication data are not available in ICD‐10‐AM, we were unable to analyze this in depth (with infliximab available in Australia since 2003 and tocilizumab since 2010). Glucocorticoids are usually required to induce rapid remission of ASD symptoms,^19^, ^30^ which makes the occurrence of osteoporosis in nearly 10% of ASD patients and early osteonecrosis in one patient noteworthy as it confirms the need for effective alternative ways to achieve early disease control. The frequency of serious infections since ASD diagnosis (27%) is slightly higher than the 21% observed by Lenert et al.^18^ and while this may partly be due to immunomodulating treatment, the fact that a considerable proportion of ASD patients also had serious infections before diagnosis lends some support to the idea of an inherent susceptibility to infection in ASD patients.^36^

The strength of this study is the reliance on a validated database with good diagnostic accuracy, reliable data linkage, long term follow up and inclusion of an age‐ and gender‐matched control group to determine differences in the main outcomes, especially serious infection and mortality. The limitations of this study relate to the fact that our data are based on a physician‐based discharge diagnosis of ASD and lack the detailed clinical and laboratory data to determine if patients fulfilled ASD classification criteria. However, a recent chart review found a 78% sensitivity for fulfilling Yamaguchi criteria in administrative hospital data, which reduces the likelihood of diagnostic error.^14^ The population‐wide capture of ASD through mandatory reporting of hospital discharge diagnoses also makes it unlikely that we have missed the rare ASD patients seen solely as outpatients. Nonetheless, the relatively small number of cases with wide confidence intervals makes it challenging to compare outcome measures and a larger (national) cohort with even longer follow up may provide additional information.

## 5 CONCLUSIONS

ASD is as uncommon in WA as in other regions. Serious infection appears to be a major risk factor. Despite high rates of chronic arthritis and joint replacement, osteoporosis and serious infections, 5‐year survival is not significantly negatively impacted. This may be because the most serious complication, hemophagocytic lymphohistiocytosis/MAS, is less common in population cohorts compared with specialist center studies.

## CONFLICT OF INTEREST

The authors report no competing interests with regards to this study.

## Supporting information


Table S1‐S2

## Data Availability

Approval for use of de‐identified data was obtained from the Human Research Ethics Committee (HREC) at the WA Department of Health (WADOH HREC# 2016.24). As this study was considered low risk by the WA Health HREC and due to the de‐identified nature of the linked health data set, the requirement for patient consent was waived. WA Health is proprietor of this administrative health data data set. Restrictions apply to the availability of these data, which were used under license of WA Health Data Linkage Branch for the current study. Data are however available from the authors upon reasonable request and after permission of WA Health and WA Data Linkage Branch.
