# Supplementary material for: Adult‐onset Still's disease in Western Australia: Epidemiology, comorbidity and long‐term outcome
Source: Int J Rheum Dis. 2022 Aug 24;25(11):1306–14. doi: 10.1111/1756-185X.14424 (PMC9805040; doi:10.1111/1756-185X.14424)
Supplement: Supplementary file 1 — Table S1‐S2 [file APL-25-1306-s001.docx]

|  | ICD-9-CM/ICD-PCS | ICD-10-AM/ACHI |
| --- | --- | --- |
| ASD | - | M06.10-M06.19 |
| Fever >39 °C ≥ 1 week | 780.60 | R50.9 |
| Arthralgia/arthritis ≥ 2 weeks | 719.40-719.49 | M25.50, M25.519, M25.529, M25.53, M25.54, M25.54, M25.54, M79.64. M25.55, M25.56, M25.57 |
| Typical rash | 782.1 | R21 |
| Leukocytosis >10,000/mm3 | 288.60-288.66, 288.69 | D72.82 |
| Sore throat | 462, 784.1 | J02.9, R07.0 |
| Lymphadenopathy | 785.6 | R59.9 |
| Hepatomegaly or splenomegaly | 789.1, 789.2 | R16.0, R16.1 |
| Abnormal liver function tests | 570, 573.3, 573.9, 790.4 | K72.00, K76.2, K71.6, K75.9, K76.9 , R74.0 |
| Smoking | 305.1, V15.82 | F17, Z72.0, Z86.43 |
| Cardiovascular disease | 390-459 | I00-I99 |
| Diabetes | 250 | E10-E14 |
| Joint replacement surgery | 81.51-81.59, 81.70-81.89 | 46300-01 to 46318.01, 48912-00 to 48924-00, 49115-00 to 49715-00 |
| Osteomyelitis | 003.24, 730.00-730.30 | M46.2, M46.3, M86.00- M87.0 |
| Immune arthritis | 446.0-447, 714.0-715,696 | M03-M09 |
| Connective tissue disease | 710.0 -711, 446.0-447 | M30 -M36 |
| Osteoarthrosis | 715.0-716 | M15.0-M20 |
| Arthrocentesis | 81.9, 83.9 | 50124 |
| Charlson comorbidity index | Am J Epidemiol 2011; 173:676-82 | |
| Serious infections | J. Rheumatology, 2020; 47:424-30 | |

Suppl Table 1 Diagnostic codes used for classifying patients and complications using ICD10-AM terminology

Suppl Table 2: Gender specific characteristics of prior medical history in ASD patients. Figures represent median values (IQR) or frequency (%)

|  | All (n=52) | Female (n=31) | Male (n=21) | P value |
| --- | --- | --- | --- | --- |
| Lookback (months) | 269 (212-314) | 252 (212-337) | 269 (194-275) | 0.45 |
| Nr prior ED visits | 171 | 92 | 79 | 0.18 |
| Nr prior admissions | 598 | 342 | 256 |  |
| Median nr admissions | 10 (4-21) | 10 (4-20.5) | 8 (5-20) | 0.56 |
| Nr with >1 admission | 41 | 25 | 14 |  |
| Prior FUO diagnosis | 11 (21) | 6 (21.4) | 5 (27.8) | 0.73 |
| Prior Rash diagnosis | 26 (50.0) | 17 (54.8) | 9 (42.8) | 0.24 |
| Prior Confirmed infection | 6 (11.5) | 3 (9.6) | 3 (14.3) | 0.16 |
| Median CCI at diagnosis | 1 (0-1) | 1 (0-1) | 0 (0-2) | 0.7 |
| m -CCI =0 | 25 (48%) | 14 (45%) | 11 (52%) | 0.05 |
| m -CCI =1 | 16 (31%) | 13 (42%) | 3 (10%) |  |
| m -CCI ≥2 | 11 (21%) | 4 (13%) | 7 (38%) |  |
|  |  |  |  |  |

In six patients ASD was the first registered hospital contact since 1980.

Suppl Figure 1: Main ICD-10AM categories for primary diagnosis in control patients during time zero admission.

Suppl Figure 2: Causes of death in ASD patients (n=6) and age/gender matched controls (n=18). Bars and numbers indicate percentages.
